# Detection of regional metabolic alteration using 7T deuterium metabolic imaging in MRI-negative, ^18^FDG-PET-positive epilepsy patients

**DOI:** 10.1007/s10334-026-01334-x

**Published:** 2026-02-19

**Authors:** Narjes Ahmadian, Romy Buijs, Mark Gosselink, igrid Otto, Kiki Tesselaar, Jeanine J. Prompers, Pieter van Eijsden, Nicole van Klink, Dennis W. Klomp, Evita C. Wiegers

**Affiliations:** 1 Center for Image Sciences, University Medical Center Utrecht, Utrecht, Netherlands; 2 Department of Neurology and Neurosurgery, Brain Center, University Medical Center Utrecht, Utrecht, Netherlands; 3 CTI Lab Support, University Medical Center Utrecht, Utrecht, Netherlands; 4 Human Biology, NUTRIM School of Nutrition and Translational Research in Metabolism, Maastricht University Medical Center+, Maastricht, Netherlands

**Keywords:** Epileptogenic region, DMI, Glutamate/Glutamine, 18FDG-PET

## Abstract

**Objective:**

Identifying presumed epileptogenic region (PER) in MRI-negative epilepsy patients is crucial for successful surgical outcomes. This study aims to determine whether dynamic deuterium metabolic imaging (DMI) at 7Tesla can detect region-specific metabolic alterations in MRI-negative, ^18^FDG-PET-positive temporal-lobe epilepsy.

**Methods:**

Five drug-resistant MRI-negative, ^18^FDG-PET-positive epilepsy patients underwent dynamic DMI. 3D ^2^H FID-MRSI scans (11:44 min each) were acquired at 7T over ~ 100 min following oral [6,6′-^2^H_2_]glucose intake. Venous plasma glucose and ^2^H glucose atom percent enrichment (APE) were measured in blood samples taken during scanning. Plasma glucose and ^2^H-glucose APE were analyzed using a General Linear Model with Repeated measures. From DMI, brain ^2^H-glucose and ^2^H glutamate/glutamine levels were analyzed using a two-level linear mixed model (factors: time and tissue type) comparing the PER and contralateral (contra-PER), hippocampus and temporal pole regions.

**Results:**

Plasma glucose and ^2^H-Glucose (Glc) atom-percent excess rose within 40 min post ingestion and then stabilized. Brain ^2^H-Glc and ^2^H-glutamate/glutamine (Glx) increased over time (p < 0.001). For ^2^H-Glc, regional differences were small, with only a modest elevation in hippocampus-PER relative to temporal-contra (p = 0.04). In contrast, ^2^H-Glx showed clear regional variation (p < 0.001), with the highest levels in hippocampus-PER, significantly exceeding PER, Temporal-PER, and Temporal-Contra (p ≤ 0.01). These findings remained consistent when averaging the final four time points.

**Conclusions:**

Ultra-high-field DMI revealed elevated hippocampal glutamatergic turnover in MRI negative epilepsy patients, with the highest levels in the epileptogenic hippocampus. These findings indicate the presence of subtle metabolic alterations in hippocampal tissue, supporting the potential of DMI to capture pathophysiological changes that remain invisible to conventional imaging.

**Supplementary Information:**

The online version contains supplementary material available at 10.1007/s10334-026-01334-x.

## Introduction

Epilepsy is a chronic neurological disorder characterized by recurrent seizures due to disrupted neuronal activity, affecting approximately 1% of the global population [1, 2]. One-third of patients are considered refractory, experiencing seizures despite treatment with antiepileptic drugs [2–4]. Temporal lobe epilepsy (TLE), often associated with mesial temporal sclerosis (MTS), accounts for a significant proportion of these cases. MTS can be reliably detected with structural MRI and is linked to favorable outcomes following surgical resection, with 70–80% achieving seizure freedom [5–8]. However, presurgical evaluation becomes particularly challenging in MRI-negative epilepsy patients; i.e., patients who lack visible abnormalities on standard MR imaging. This is the case in approximately one-third of the epilepsy patients.

Identifying the brain area that causes seizures (i.e. the presumed epileptogenic region (PER)) remains the cornerstone of successful epilepsy surgery [7, 8]. MRI-negative patients often require additional testing to localize the PER, including invasive intracranial electroencephalography (EEG) recordings, magnetoencephalography (MEG), Single Photon Emission Computed Tomography (SPECT), ultra-high field MRI or high-density EEG. Since altered metabolism is thought to be a hallmark of epileptic tissue, imaging methods that probe metabolic activity are of particular interest. ^18^F-fluorodeoxyglucose positron emission tomography (^18^FDG-PET) is often employed to identify interictal hypometabolic regions that may correspond to the PER [9].

To capture metabolic alterations beyond glucose uptake as measured with ^18^FDG-PET, attention has turned to glutamate and glutamine, key metabolites in neurotransmission and strongly implicated in epilepsy [2, 8, 10]. Studies using ^1^H magnetic resonance spectroscopy (MRS) and glutamate chemical exchange saturation transfer (GluCEST) demonstrated elevated glutamate or combined glutamate/glutamine (Glx) levels in the hippocampus of temporal lobe epilepsy (TLE) patients, especially in those without hippocampal sclerosis (HS) [4–11]. However, other studies have reported either a decrease or no significant difference in glutamate or Glx levels when comparing epileptogenic tissue to the contralateral hemisphere or healthy controls [12, 13]. A fundamental limitation of ^1^H MRS and GluCEST is that these techniques measure metabolite concentrations rather than metabolic flux, potentially masking changes in neurotransmitter cycling. Ex vivo ^13^C MRS studies of neurosurgical specimens have indeed highlighted the importance of assessing glutamate metabolism in epilepsy, demonstrating a marked reduction in glutamate cycling within epileptogenic compared to histologically normal tissue [14]. ^13^C MRS can be used in vivo to assess metabolism, it is costly and typically limited to imaging a single large voxel, necessitating prior localization of the epileptogenic zone.

Deuterium metabolic imaging (DMI) is a novel, non-invasive metabolic imaging technique based on magnetic resonance spectroscopic imaging (MRSI), which uses deuterium (^2^H)-labeled substrates to track metabolic pathways in vivo. Following oral administration of ^2^H-labeled glucose, DMI can visualize the metabolic conversion into its downstream products like Glx and lactate [15]. Compared to ^13^C MRS, DMI benefits from higher sensitivity due to favorable relaxation times and reduced susceptibility to magnetic field inhomogeneities, particularly at ultra-high field strengths [15]. These features allow dynamic tracking of glycolytic and TCA (tricarboxylic acid) cycle activity and enable 3D metabolic mapping of the brain [16].

Whole brain metabolic mapping with DMI enables region-specific analysis. Next to the PER, other areas of the brain are thought to contribute to seizure generation, propagation or compensation. Temporal regions, including the hippocampus, amygdala, and uncus, are particularly important due to their roles in seizure initiation and propagation in temporal lobe epilepsy, making them frequent targets in epilepsy research [17–19]. Here we use DMI at ultra-high field strength to quantify regional dynamics of ^2^H-glc and ^2^H-Glx in MRI-negative, ^18^FDG-PET-positive TLE patients. By visualizing region-specific metabolic alterations, our goal is to detect regional metabolic alterations in challenging non-lesional epilepsy cases.

## Methods

### Experimental protocol

We selected five adult epilepsy patients in pre-surgical workup to epilepsy surgery, with clinically defined temporal lobe epilepsy based on semiology and video electroencephalography. The patients had no structural abnormalities on clinical 3T MRI and showed hypometabolism on clinical ^18^FDG-PET (Table 1).

**Table 1:** Patients characteristics

Patient	Sex	Age (years)	BMI (kg/m^2^)	Fasting plasma Glucose level (mM/L)	PER location
Epi-1	M	27	27	4.3	L. mesiotemporal lobe
Epi-2	F	47	33	4.5	R. mesiotemporal lobe
Epi-3	M	45	31	4.8	R. parietal lobe
Epi-4	F	45	17	3.8	L. mesiotemporaal lobe
Epi-5	F	33	22	3.5	L. mesiotemporaal lobe

*M* male, *L* left, *R* right, PER epileptogenic zone

The study received ethical approval from the NedMec Medical Ethics Committee (NL83501.041.23) in Utrecht, the Netherlands. All procedures were conducted in accordance with the ethical principles outlined in the Declaration of Helsinki (1964) and its subsequent revisions. Written informed consent was obtained from all participants prior to their inclusion in the study.

### DMI hardware and acquisition

DMI data were collected using a 7T MR system (Philips, Best, NL), featuring a home-built deuterium (^2^H) transmit coil integrated behind the bore of the scanner (60 cm diameter, 40 cm length) [20]. For signal reception, a head coil was used, consisting of eight trapezoid-shaped ^2^H receive loops (seven measuring 200 × 75–120 mm and one smaller frontal loop of 120 × 75–180 mm, all with 6 mm copper traces). This setup was combined with eight ^1^H transmit/receive dipole antennas [21], positioned at least 35 mm away from the ^2^H loops to minimize interference.

After the participants were placed in the scanner, B_0_ shimming was conducted, and both a T_1_-weighted image and a baseline DMI scan were acquired. Then, patients consumed 0.50 g/kg body weight of [6,6′-^2^H_2_]glucose dissolved in water (0.2 g/ml) within ~ 3-4 min [16] via a 1.5 m tube while they remained in the MR scanner. DMI data collection continued right after [6,6′-^2^H_2_]glucose consumption and lasted for approximately 100–120 min, depending on patient’s compliance.

DMI data was acquired using a hamming-weighted 3D FID-MRSI sequence with a nominal voxel size of 12 × 12 × 12 mm^3^ (1.73 ml). Because of the acquisition-weighted spatial response function, this corresponds to an effective spherical volume of 3.84 ml, calculated following the methods described by Pohmann et al. [22]. Additional acquisition parameters were field of view (FOV): 240 × 180 × 216 mm^3^, repetition time (TR):100 ms, echo time (TE):1.82 ms, spectral bandwidth: 2800 Hz, 256 data points, four sample averages at k-space center, acquisition time: 11:44 min per scan).

### Blood sampling and analysis

Participants fasted overnight and were scanned in the morning. Participants had an intravenous catheter placed in the median cubital vein. 5 ml blood samples were taken from the intravenous catheter every 10 min.

Plasma glucose levels were measured with a YSI glucose analyzer (2500 series, YSI, USA), and deuterium atom percent excess (APE) in plasma glucose was determined by gas chromatography-mass spectrometry [16, 32–34].

### Post-processing

Data processing was done using in-house MATLAB scripts (Matlab R2021a, MathWorks, USA), including spatial Fourier transformation and phase correction. Data from the eight ^2^H receive channels were combined via Whitened Singular-Value Decomposition (WSVD) [23]. Following coil combination, the data underwent PCA-based denoising at each time point using a 5 × 5 × 5 patch size [24], after which a 5 Hz exponential apodization and spectral zero-filling to 2048 points were applied [25]. Signals from deuterated water (HDO), ^2^H-glucose (^2^H-Glc), and ^2^H-Glx were fitted with AMARES using Lorentzian line shapes [26], in two steps as described previously [27]. Only voxels in which the HDO signal from the baseline DMI scan (i.e., prior to [6,6-^2^H₂]-glucose administration) had a Cramèr–Rao Lower Bound (CRLB) below 10% were included in the analysis for all subsequent datasets. For ^2^H-Glc, ^2^H-Glx, and ^2^H-Lac, a CRLB threshold of 50% was applied at each individual time point. All signals were normalized voxel-wise to baseline HDO signal amplitude, assuming a HDO concentration of 17.2 mM. Signals were corrected for water content (grey matter: 0.78, white matter: 0.65, CSF: 0.97), number of deuterons, label loss, and T_1_-relaxation times [28–30].

In the ~ 1–6 months prior to the DMI scan, each patient underwent an ^18^FDG-PET scan as part of standard clinical care. The suspected epileptogenic region was defined as the hypometabolic region in each patient’s ^18^FDG-PET scan, manually delineated by an experienced ^18^FDG-PET interpreter. The ^18^FDG-PET was co-registered to the T_1_-weighted 7T image using SPM12. Average metabolite concentrations per timepoint were calculated in the PER and contralateral healthy tissue (contra-PER), and in two brain regions commonly involved in TLE: the hippocampus and temporal pole [31]. Anatomical masks for these regions were generated using AAL, Talairach Daemon, and IBA SPM 116 atlases in SPM12, then transformed from MNI to native space, split by hemisphere, and resampled to match DMI resolution. A voxel was assigned to a specific ROI if at least 25% of its volume overlapped with corresponding resampled mask. Additionally, mean ^18^FDG-PET uptake and mean metabolite concentrations per ROI were calculated over the final 4 timepoints per patient. This analysis of metabolite concentrations in the hippocampus and temporal pole was also performed on data of 5 previously published healthy volunteers (3 males/2 females, mean age 29 ± 7.8 years) (16), acquired using the exact same scanning protocol.

Only for visualization, the metabolic maps were upscaled per slice by a factor of five using bicubic interpolation, followed by Gaussian smoothing (σ = 1.2).

### Statistical analysis

Mean plasma ^2^H-Glc APE and mean plasma glucose concentrations of [6,6′-^2^H_2_]glucose ingestion were calculated in all patients. The serial plasma glucose and plasma ^2^H-Glc APE were compared between patients over the entire scan duration using a General Linear Model with Repeated measures. To assess individual variability, a linear mixed model was used with patient as a random effect.

Statistical significance of time and brain ROI (PER, contra-PER, Hippocampus-PER, Hippocampus-contra, Temporal-PER, Temporal-contra) on brain ^2^H-Glc and brain ^2^H-Glx levels were analyzed using a two level Linear Mixed Model, with time and tissue type as fixed factors. For time as a factor, the dynamic changes in brain metabolite concentrations were compared in all tissue types. For the tissue type as a factor, the mean concentration of brain metabolites over all time points were compared between the different tissues. Average metabolite levels over the final 4 timepoints and ^18^FDG-PET uptake were compared between ROIs with ANOVA. ^18^FDG-PET uptake in PER vs. contra-PER was compared with a paired t-test.

If the results of the tests were significant, Bonferroni Post-Hoc tests were conducted.

Statistical significance was set at p < 0.05 for the above tests using IBM SPSS Statistics (version 27).

## Results

Among the five included patients, four showed hypometabolism in the temporal lobe on ^18^FDG-PET, consistent with the clinical diagnosis of temporal lobe epilepsy. In one patient, however, PET imaging revealed a parietal hypometabolic region (Table 1). Despite this discrepancy, the patient was included in the study given the clinical semiology strongly suggestive of temporal lobe epilepsy. Mean ^18^FDG-PET uptake in the PER was −5.3 ± 3.8% lower (p = 0.04) compared to contra-PER.

**Fig. 1:**
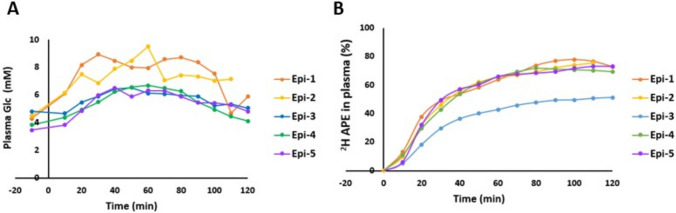
**A** Plasma glucose concentration and **B** Plasma ^2^H-Glc APE following oral consumption (at T = 0 min) of [6,6′-^2^H_2_]glucose

Plasma glucose levels and ^2^H-Glc APE initially rose in the first 40 min after [6,6′-^2^H_2_]glucose ingestion, stabilizing between 40 and 120 min for ^2^H-Glc APE (Fig. 1B). For both measures, a repeated measures ANOVA showed a significant effect of time, indicating a change over the course of the experiment (p < 0.001). The analysis for the individual variability revealed significant differences between patients in average levels of both plasma glucose and APE (p < 0.001).

**Fig. 2:**
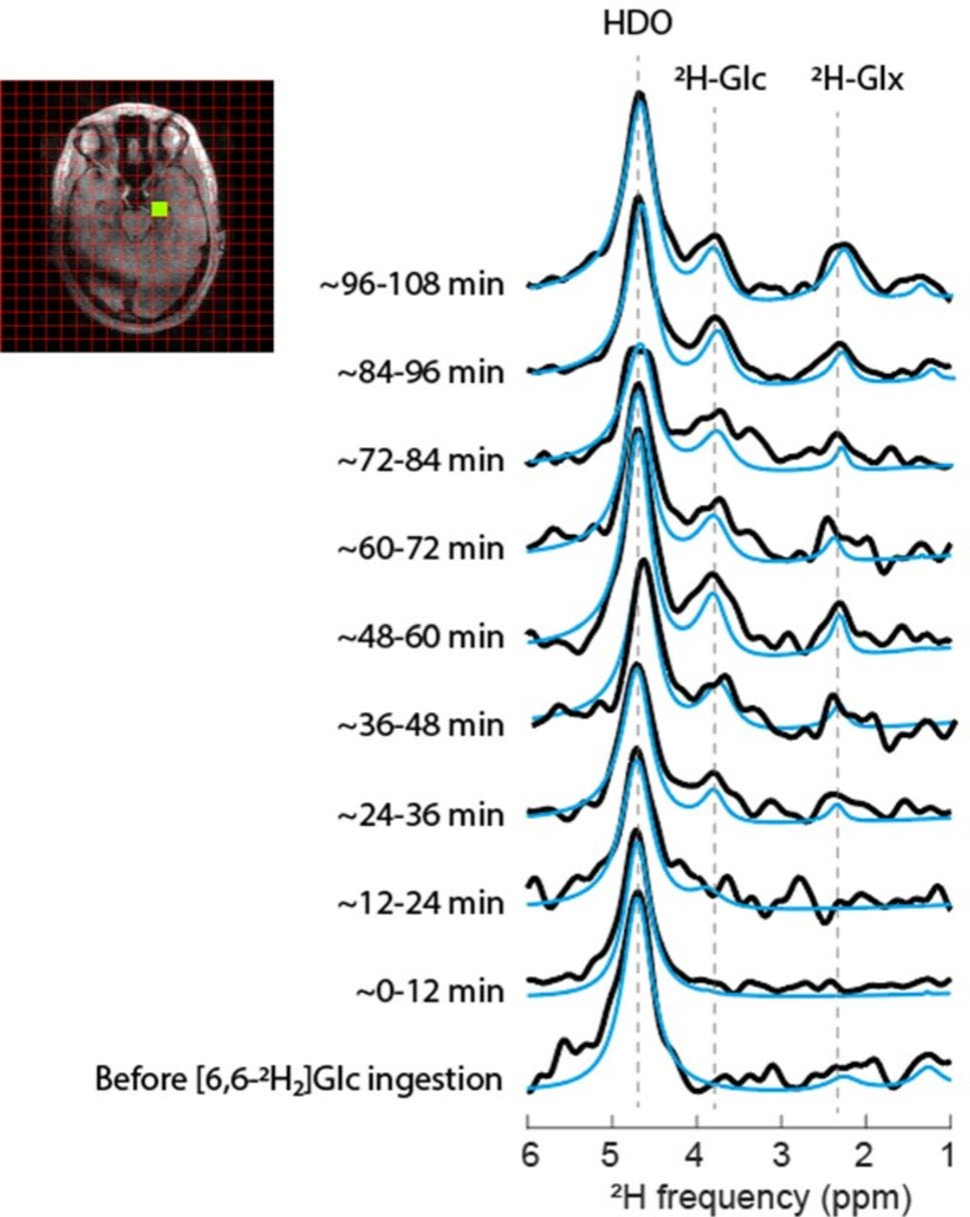
^2^H MR spectra (in black) and corresponding fits (in blue) from one voxel in the PER of one subject (EPI-1) as a function of time. The voxel location is indicated by the green box on the T1-weighted scan. The first spectrum is acquired prior to [6,6-^2^H_2_]glucose ingestion. Dashed lines mark the resonance frequencies of HDO, ^2^H-Glc, and ^2^H-Glx

**Fig. 3:**
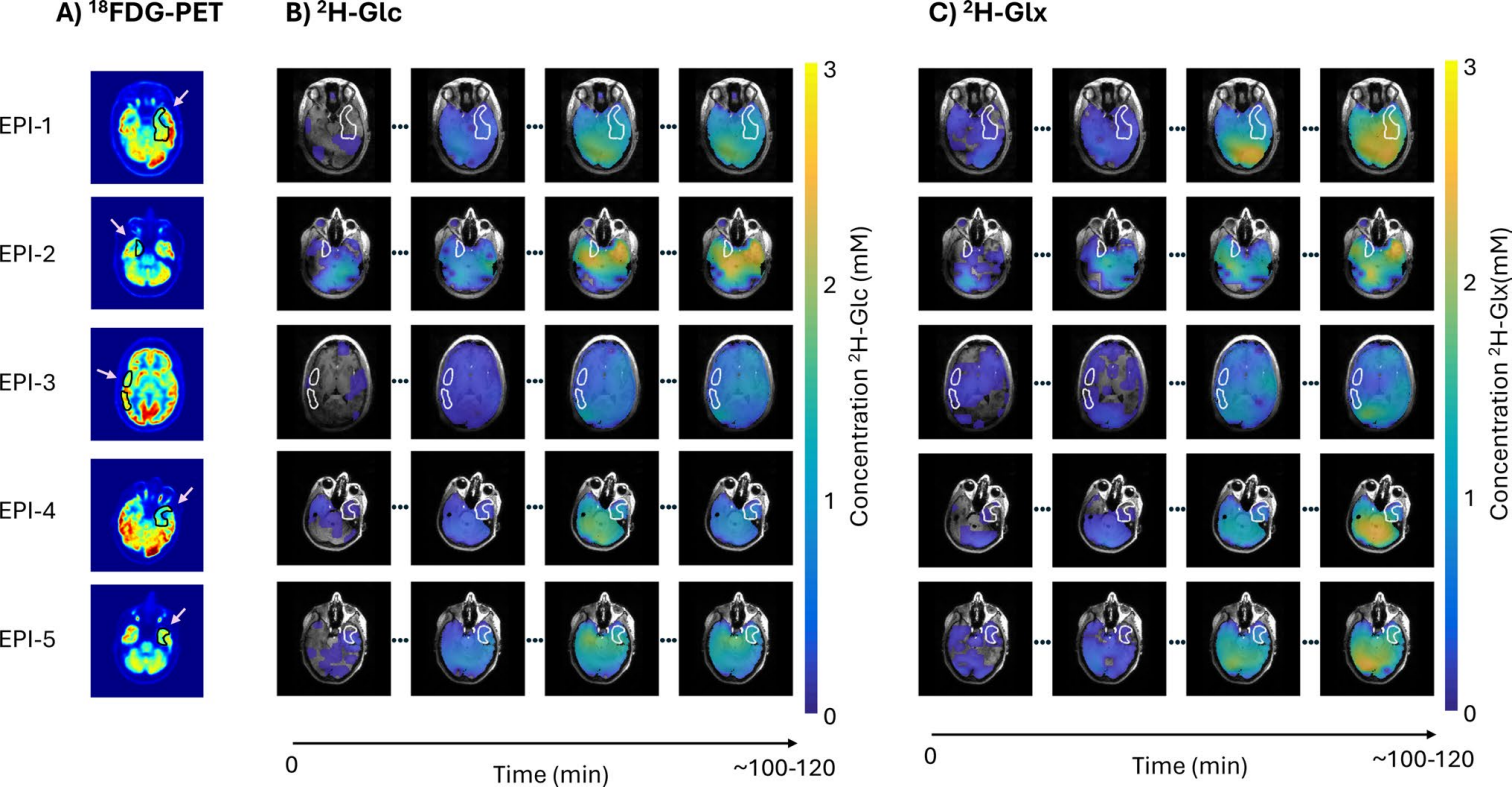
**A** ^18^FDG-PET scans and Dynamic DMI maps of **B** ^2^H-Glc and **C** ^2^H-Glx after oral consumption of [6,6′-^2^H_2_]glucose, per subject (Epi-1 to Epi-5). Only 4 timepoints are shown here. DMI maps are in the same slice as the ^18^FDG-PET scan. The presumed epileptogenic region (PER)) is indicated by the outlined areas and the pink arrow in **A**. Color bars indicate metabolite concentrations (in mM)

**Fig. 4:**
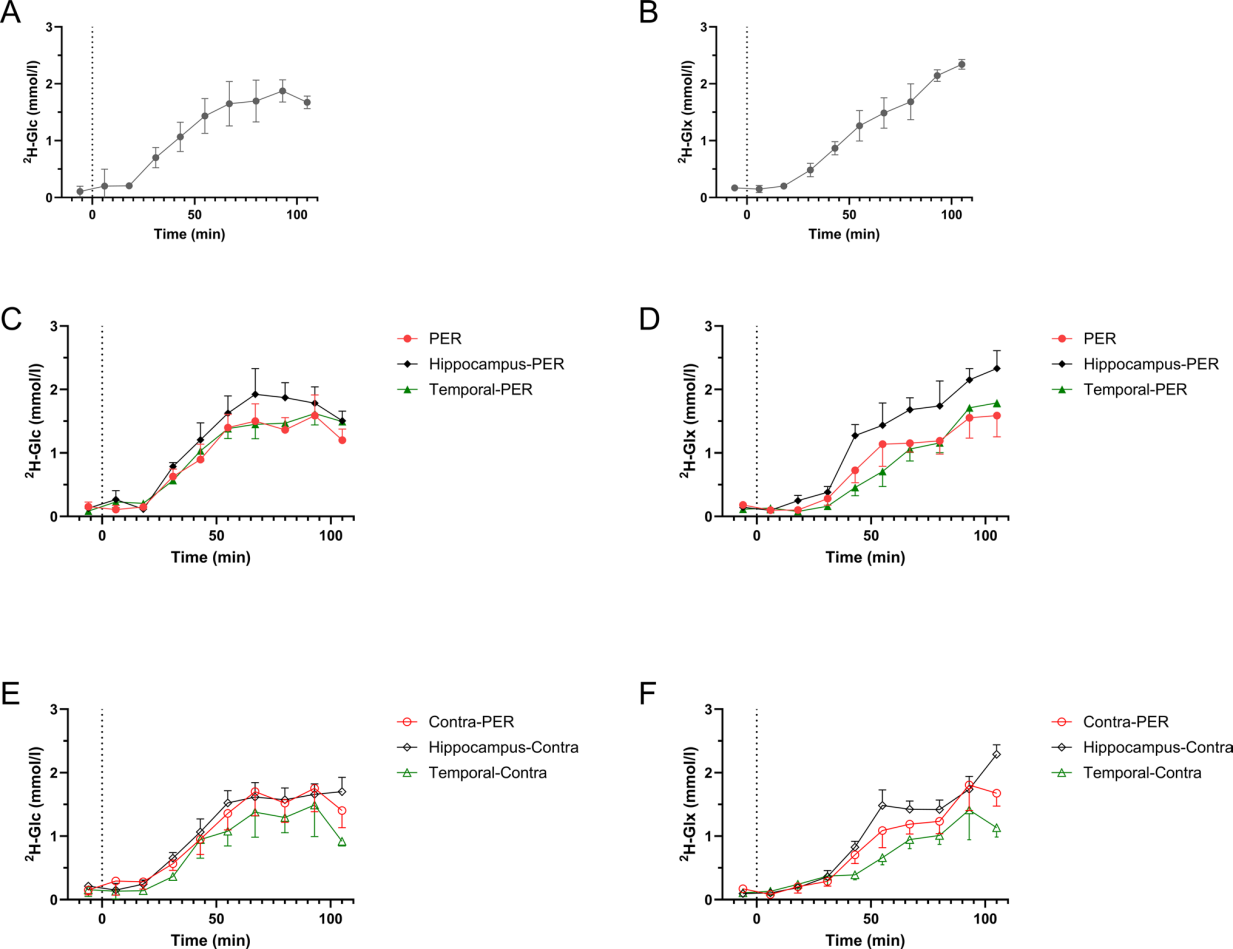
Time curves showing mean concentrations and standard deviations (error bars) of ^2^H-labeled brain metabolites following oral intake of [6,6′-^2^H₂]glucose at t = 0 min, across all patients. **A** Whole-brain ^2^H-Glc concentration over time. **B** Whole-brain ^2^H-Glx concentration over time. **C** Regional ^2^H-Glc concentrations for PER, hippocampus-PER, and temporal-PER. **D** Regional ^2^H-Glx concentrations for PER, hippocampus-PER, and temporal-PER. **E** Regional ^2^H-Glc concentrations for contra-PER, hippocampus-contra, and temporal-contra. **F** Regional ^2^H-Glx concentrations for contra-PER, hippocampus-contra, and temporal-contra

Representative dynamic ^2^H spectra of 1 subject are shown in Fig. 2. Figure 3 displays a subset of the timepoints of the dynamic DMI maps (DMI maps of all timepoints are available in Supplementary Fig. 1 and 2). Figure 4 shows the concentration curves of brain ^2^H-Glc and ^2^H-Glx following the oral consumption of [6,6′-^2^H_2_]glucose. The results revealed a significant effect of time on brain ^2^H-Glc levels (p < 0.001), indicating that ^2^H-Glc concentrations changed significantly over the course of the dynamic acquisition. There was also a significant main effect of tissue type (p = 0.039), reflecting regional variation in ^2^H-Glc. Estimated marginal means were highest in hippocampus-PER (1.12), followed by hippocampus-contra (1.04), contra-PER (1.01), temporal-PER (0.94), PER (0.90) and temporal-contra (0.83). Pairwise comparisons revealed that hippocampus-PER showed significantly higher ^2^H-Glc levels than temporal-contra (p = 0.04), whereas no other pairwise tissue comparisons reached significance (all p ≥ 0.332), including PER versus contra-PER. Thus, overall glucose levels were broadly comparable across regions, with a modest but significant elevation of ^2^H-Glc in hippocampus-PER relative to the contralateral temporal lobe.

For brain ^2^H-Glx, both time (p < 0.001) and tissue type (p < 0.001) had significant main effects, while the interaction between time and tissue was not significant (p = 0.91), indicating similar temporal dynamics across regions. Hippocampus-PER exhibited the highest mean ^2^H-Glx level (1.10), followed by hippocampus-contra (0.96), contra-PER (0.84), PER (0.78), temporal-PER (0.71) and temporal-contra (0.66). Pairwise comparisons showed that hippocampus-PER had significantly higher ^2^H-Glx than PER (p = 0.004), temporal-PER (p < 0.001) and temporal-contra (p < 0.001). In addition, hippocampus-contra showed significantly higher ^2^H-Glx than temporal-contra (p = 0.010). Differences between hippocampus-PER and hippocampus-contra, and between hippocampus-PER and contra-PER, did not reach significance after correction. Together, these findings indicate a regionally specific elevation of ^2^H-Glx within the hippocampal formation, most pronounced in hippocampus-PER.

**Fig. 5:**
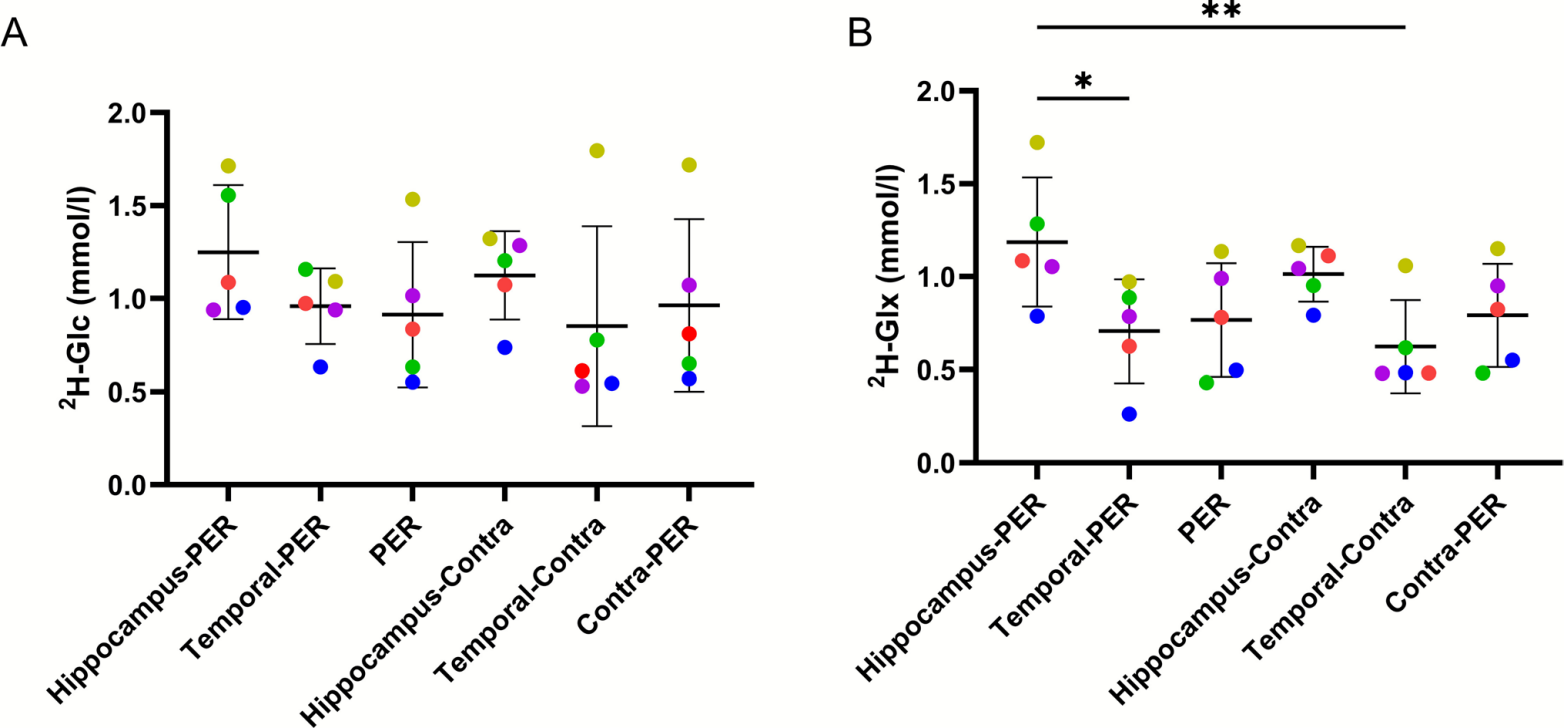
Mean brain ^2^H-Glc (**A**) and ^2^H-Glx (**B**) levels over the final 4 timepoints per patients. Concentrations are presented for, epileptogenic zone (PER), hippocampus, and temporal pole regions. (and corresponding contralateral regions. Different colors represent individual patients. *p < 0.05; **p < 0.01

When average metabolite levels over the final four time points were compared across patients, results were consistent: brain ^2^H-Glc levels showed no significant regional differences (p = 0.21; Fig. 5A), whereas ^2^H-Glx levels were significantly elevated in the hippocampus-PER relative to Temporal-PER and Temporal-Contra (p = 0.008; Fig. 5B). No significant differences were found in ^18^FDG-PET uptake between these ROIs (Supplementary Fig. 3). In healthy volunteers, there was no difference in ^2^H-Glc levels in the hippocampus compared to the temporal pole. ^2^H-Glx levels were numerically higher in the hippocampus, but this difference did not reach statistical significance (Supplementary Fig. 4).

## Discussion

This study examined regional brain metabolism in MRI-negative, ^18^FDG-PET-positive epilepsy patients using dynamic deuterium metabolic imaging (DMI). By tracking ^2^H-labeled glucose and its downstream metabolites, we aimed to evaluate the metabolic distinction between the PER, contralateral healthy brain tissue, Hippocampus-PER, Hippocampus-contra, Temporal-PER and Temporal-contra.

^2^H-Glx levels were significantly elevated in the hippocampus-PER compared to the temporal poles. The hippocampus is known to have a high density of NMDA receptors, making it particularly vulnerable to glutamate-driven injury [34]. This regional specificity suggests an upregulated glutamatergic metabolism or enhanced glutamate turnover within epileptogenic hippocampal tissue. The observed high flux of glutamate in both hippocampus may be the result of overstimulated glutamate receptors, triggering a chain reaction of excitation within glutamatergic networks [35–37]. In healthy volunteers and the contralateral hippocampus in the epilepsy patients, we found numerically but not statistical significant elevated ^2^H-Glx levels. These findings, observed in our small cohort of subjects, support the notion that the elevation of ^2^H-Glx may be indeed specific to the hippocampus-PER.

All subjects in this study showed lower ^18^FDG-PET uptake in the PER, which is expected, especially during the interictal phase [32].Despite this, we observed no significant difference in ^2^H-Glc concentrations between PER and contra-PER. This suggests that glucose uptake and its initial metabolism may remain relatively intact in the PER, or that DMI, at its current spatial resolution, may not detect the subtle changes in glucose uptake as sensitively as ^18^FDG-PET.

Several factors may contribute to this discrepancy between ^18^FDG-PET uptake and ^2^H-Glc levels. First, the relatively large voxel size in DMI (12 × 12 × 12 mm) likely includes both epileptogenic and surrounding healthy tissue, leading to signal averaging that reduces the sensitivity to small focal abnormalities. Second, the spatial and temporal patterns of glucose metabolism in epilepsy are complex. While ^18^FDG-PET measures steady-state glucose uptake (mainly reflecting glycolytic activity in astrocytes), DMI detects downstream oxidative metabolism, including the conversion of labeled glucose to glutamate and glutamine via the TCA cycle. Consequently, regions showing hypometabolism on ^18^FDG-PET may still exhibit elevated oxidative turnover or compensatory activity. Thus, rather than a contradiction, the observed elevation of hippocampal ^2^H-Glx highlights that DMI and ^18^FDG -PET provide distinct yet complementary insights into cerebral glucose utilization [9].

Our study also confirmed that plasma glucose levels and deuterium atom percent excess (APE) increased as expected after oral [6,6′-^2^H₂]glucose ingestion, validating the systemic uptake and labeling necessary for DMI. While APE plateaued after 40 min, brain ^2^H-Glc and ^2^H-Glx continued to rise, reflecting ongoing cerebral uptake and metabolism. This dynamic data acquisition is a key advantage of DMI, providing insight into metabolic flux over time [28].

It is important to note that the patients in this study were MRI-negative, meaning no structural lesions were visible on standard imaging, and ^18^FDG-PET abnormalities in these patients are very subtle. Therefore, metabolic abnormalities detectable by DMI could differ significantly from MRI-positive epilepsy patients, who typically have pronounced structural lesions accompanied by more distinct metabolic disruptions. Future comparative studies including both MRI-negative and MRI-positive patient cohorts could clarify how structural pathology influences metabolic imaging results.

Despite promising findings, this study has several limitations. The small patient cohort (n = 5) limits generalizability, and individual variability in metabolism, and medication could have influenced the results. Furthermore, the interictal phase may not be the most metabolically active state of the PER. Future studies should investigate postictal or ictal phases, which may reveal more distinct metabolic signatures, although this is practically challenging. Improved spatial resolution through advanced coil designs, acceleration techniques, or postprocessing could also enhance regional specificity.

## Conclusion

While brain metabolism measured with DMI did not differentiate the PER from other regions in MRI-negative epilepsy patients, glutamate/glutamine metabolism showed clear elevations within the hippocampus, with the highest levels in the epileptogenic hippocampus. This suggests that DMI can capture hippocampal-specific metabolic alterations relevant to temporal lobe epilepsy, although the lack of differences between epileptogenic and contralateral hippocampus indicates that further validation is needed. Future studies with higher-resolution DMI, and exploration of different epileptic phases are required to determine whether these hippocampal metabolic changes can reliably contribute to presurgical evaluation.

## Supplementary Information

Below is the link to the electronic supplementary material.

Dynamic DMI maps of 2H-Glc after oral consumption of [6,6′-^2^H_2_]glucose, per subject (Epi-1 to Epi-5), including of all timepoints. The presumed epileptogenic region (PER) is indicated by the outlined areas. Color bars indicate metabolite concentrations (in mM). Supplementary file1 (PNG 4791 KB)

Dynamic DMI maps of 2H-Glx after oral consumption of [6,6′-^2^H_2_]glucose, per subject (Epi-1 to Epi-5), including of all timepoints. The presumed epileptogenic region (PER) is indicated by the outlined areas. Color bars indicate metabolite concentrations (in mM). Supplementary file2 (PNG 4818 KB)

Mean ^18^FDG-PET uptake values across patients for the epileptogenic region (PER), hippocampus, and temporal pole, along with their contralateral side. Each dot represents an individual patient, with horizontal bars indicating group means and error bars showing standard deviations. Supplementary file3 (PNG 64 KB)

Mean brain ^2^H-Glc (A) and ^2^H-Glx (B) levels over the final four time points in healthy volunteers. Metabolite concentrations are shown for the left and right hippocampus and temporal pole. Individual colors represent separate volunteers. Supplementary file4 (PNG 232 KB)

## Data Availability

The datasets generated and analyzed during this study are not publicly accessible owing to ethical considerations. Participants did not provide consent for open data sharing, and full anonymization of the data is not feasible.

## References

[1] Fisher RS, Acevedo C, Arzimanoglou A, Bogacz A, Cross JH, Elger CE et al (2014) ILAE official report: a practical clinical definition of epilepsy. Epilepsia 55(4):475–482. 10.1111/epi.12550 24730690 10.1111/epi.12550

[2] Sarlo GL, Holton KF (2021) Brain concentrations of glutamate and GABA in human epilepsy: a review. Seizure 91:213–227. 10.1016/j.seizure.2021.06.028 34233236 10.1016/j.seizure.2021.06.028

[3] Hadar PN, Kini LG, Nanga RPR, Shinohara RT, Chen SH, Shah P et al (2021) Volumetric glutamate imaging (GluCEST) using 7T MRI can lateralize nonlesional temporal lobe epilepsy: a preliminary study. Brain Behav 11(8):e02164. 10.1002/brb3.2164 34255437 10.1002/brb3.2134 PMC8413808

[4] Davis KA, Nanga RPR, Das S, Chen SH, Hadar PN, Pollard JR et al (2015) Glutamate imaging (GluCEST) lateralizes epileptic foci in nonlesional temporal lobe epilepsy. Sci Transl Med 7(309):309ra161. 10.1126/scitranslmed.aab3430 26468323 10.1126/scitranslmed.aaa7095 PMC4710355

[5] Woermann FG, McLean MA, Bartlett PA, Parker GJ, Barker GJ, Duncan JS (1999) Short echo time single-voxel 1H MRS in MRI-negative temporal lobe epilepsy. Epilepsia 40(7):806–812. 10.1111/j.1528-1157.1999.tb00805.x 10.1002/1531-8249(199903)45:3<369::aid-ana13>3.0.co;2-q 10072052

[6] Simister RJ, Woermann FG, McLean MA, Bartlett PA, Barker GJ, Duncan JS (2002) A short-echo-time proton magnetic resonance spectroscopic imaging study of temporal lobe epilepsy. Epilepsia 43(9):1021–1031. 10.1046/j.1528-1157.2002.07402.x 12199727 10.1046/j.1528-1157.2002.50701.x

[7] Simister RJ, McLean MA, Salmenpera TM, Barker GJ, Duncan JS (2008) The effect of epileptic seizures on proton MRS-visible neurochemical concentrations. Epilepsy Res 81(1):36–43. 10.1016/j.eplepsyres.2008.04.004 18508239 10.1016/j.eplepsyres.2008.04.009

[8] Çavuş I, Romanyshyn JC, Kennard JT, Farooque P, Williamson A, Eid T et al (2016) Elevated basal glutamate and unchanged glutamine and GABA in refractory epilepsy: microdialysis study of 79 patients. Ann Neurol 80(1):35–45. 10.1002/ana.24685 27129611 10.1002/ana.24673

[9] Pfund Z, Chugani DC, Juhász C, Muzik O, Chugani HT, Wilds IB, Moore GJ (2000) Evidence for coupling between glucose metabolism and glutamate cycling using FDG PET and ^1^H-MRS in patients with epilepsy. J Cereb Blood Flow Metab 20(5):871–878. 10.1097/00004647-200005000-00014 10826538 10.1097/00004647-200005000-00014

[10] Eid T, Thomas MJ, Spencer DD, Runden-Pran E, Lai JC, Malthankar GV, Ottersen OP (2004) Loss of glutamine synthetase in the human epileptogenic hippocampus: possible mechanism for raised extracellular glutamate in mesial temporal lobe epilepsy. Lancet 363(9402):28–37 14723991 10.1016/s0140-6736(03)15166-5

[11] Riederer F, Bittšansky M, Schmidt C, Mlynárik V, Baumgartner C, Moser E et al (2006) 1H MRS at 3T in cryptogenic and mesial temporal lobe epilepsy. NMR Biomed 19(5):544–553. 10.1002/nbm.1052 16521092 10.1002/nbm.1029

[12] Doelken MT, Stefan H, Pauli E, Stadlbauer A, Struffert T, Engelhorn T et al (2008) 1H-MRS profile in MRI positive- versus MRI negative patients with temporal lobe epilepsy. Seizure 17(6):490–497. 10.1016/j.seizure.2008.01.007 18337128 10.1016/j.seizure.2008.01.008

[13] Pan JW, Venkatraman TN, Vives KP, Spencer DD (2006) Quantitative glutamate spectroscopic imaging of the human hippocampus. NMR Biomed 19(2):209–216. 10.1002/nbm.1019 16479532 10.1002/nbm.1019 PMC3657732

[14] Petroff OA, Errante LD, Rothman DL, Kim JH, Spencer DD (2002) Glutamate-glutamine cycling in the epileptic human hippocampus. Epilepsia 43(7):703–710. 10.1046/j.1528-1157.2002.38901.x 12102672 10.1046/j.1528-1157.2002.38901.x

[15] De Feyter HM, Behar KL, Corbin ZA, Fulbright RK, Brown PB, McIntyre S, Nixon TW, Rothman DL, de Graaf RA (2018) Deuterium metabolic imaging (DMI) for MRI-based 3D mapping of metabolism in vivo. Sci Adv 4(8):eaat7314. 10.1126/sciadv.aat7314 30140744 10.1126/sciadv.aat7314 PMC6105304

[16] Ahmadian N, Konig MM, Otto S, Tesselaar K, van Eijsden P, Gosselink M, Gursan A, Klomp DW, Prompers JJ, Wiegers EC (2024) Human brain deuterium metabolic imaging at 7 T: impact of different [6,6′-2H2]glucose doses. J Magn Reson Imaging. 10.1002/jmri.29532 39058248 10.1002/jmri.29532 PMC11803682

[17] Spencer S, Spencer D (1994) Entorhinal-hippocampal interactions in medial temporal lobe epilepsy. Epilepsia 35:721–727 8082614 10.1111/j.1528-1157.1994.tb02502.x

[18] Bartolomei F, Chauvel P, Wendling F (2008) Epileptogenicity of brain structures in human temporal lobe epilepsy: a quantified study from intracerebral EEG. Brain 131(7):1818–1830. 10.1093/brain/awn111 18556663 10.1093/brain/awn111

[19] Jack CR Jr et al (1990) Temporal lobe seizures: lateralization with MR volume measurements. Radiology 175(2):423–429. 10.1148/radiology.175.2.2326460 2183282 10.1148/radiology.175.2.2183282

[20] Gursan A, Kahraman-Agir B, Gosselink M, Welting D, Froeling M, Hoogduin H, Wiegers EC, Prompers JJ, Klomp DWJ (2025) Development of a double tuned 2H/31P whole-body birdcage transmit coil for 2H and 31P MR applications from head to toe at 7 T. NMR Biomed. 38(3):e5325. 10.1002/nbm.5325 39888087 10.1002/nbm.5325 PMC11783138

[21] Raaijmakers AJ, Italiaander M, Voogt IJ, Luijten PR, Hoogduin JM, Klomp DW, van den Berg CA (2016) The fractionated dipole antenna: a new antenna for body imaging at 7 Tesla. Magn Reson Med 75(3):1366–1374 25939890 10.1002/mrm.25596

[22] Pohmann R, von Kienlin M (2001) Accurate phosphorus metabolite images of the human heart by 3D acquisition-weighted CSI. Magn Reson Med 45(5):817–826. 10.1002/mrm.1110 11323808 10.1002/mrm.1110

[23] Rodgers CT, Robson MD (2010) Receive array magnetic resonance spectroscopy: whitened singular value decomposition (WSVD) gives optimal Bayesian solution. Magn Reson Med 63(4):881–891 20373389 10.1002/mrm.22230

[24] Froeling M, Prompers JJ, Klomp DW, van der Velden TA (2021) PCA denoising and Wiener deconvolution of 31P 3D CSI data to enhance effective SNR and improve point spread function. Magn Reson Med 85(6):2992–3009. 10.1002/mrm.28654 33522635 10.1002/mrm.28654 PMC7986807

[25] Veraart J, Novikov DS, Christiaens D, Ades-Aron B, Sijbers J, Fieremans E (2016) Denoising of diffusion MRI using random matrix theory. Neuroimage 142:394–406 27523449 10.1016/j.neuroimage.2016.08.016 PMC5159209

[26] Purvis LAB, Clarke WT, Biasiolli L, Valkovič L, Robson MD, Rodgers CT (2017) OXSA: an open-source magnetic resonance spectroscopy analysis toolbox in MATLAB. PLoS ONE 12(9):e0185356 28938003 10.1371/journal.pone.0185356 PMC5609763

[27] Ahmadian N, Gosselink M, Otto S, Welting D, Tesselaar K, Snijders T, van Eijsden P, Prompers J, Klomp D, Wiegers E (2025) Dynamic deuterium metabolic imaging in glioblastoma at 7T. MAGMA. 10.1007/s10334-025-01299-3 41066028 10.1007/s10334-025-01299-3 PMC13354616

[28] Kaggie JD, Khan AS, Matys T, Schulte RF, Locke MJ, Grimmer A et al (2022) Deuterium metabolic imaging and hyperpolarized (13)C-MRI of the normal human brain at clinical field strength reveals differential cerebral metabolism. Neuroimage 257:119284 35533826 10.1016/j.neuroimage.2022.119284

[29] Niess F, Strasser B, Hingerl L, Niess E, Motyka S, Hangel G et al (2023) Reproducibility of 3D MRSI for imaging human brain glucose me-tabolism using direct ((2)H) and indirect ((1)H) detection of deuterium labeled compounds at 7T and clinical 3T. Neuroimage 277:120250 37414233 10.1016/j.neuroimage.2023.120250 PMC11019874

[30] de Graaf RA, Thomas MA, Behar KL, De Feyter HM (2021) Characterization of kinetic isotope effects and label loss in deuterium-based isotopic labeling studies. ACS Chem Neurosci 12(1):234–243 33319987 10.1021/acschemneuro.0c00711 PMC9890388

[31] Allone C, Buono VL, Corallo F, Pisani LR, Pollicino P, Bramanti P et al (2017) Neuroimaging and cognitive functions in temporal lobe epilepsy: a review of the literature. J Neurol Sci 381:7–15 28991719 10.1016/j.jns.2017.08.007

[32] Macallan DC, Asquith B, Zhang Y, de Lara C, Ghattas H, Defoiche J, Beverley PC (2009) Measurement of proliferation and disappearance of rapid turnover cell populations in human studies using deuterium-labeled glucose. Nat Protoc 4(9):1313–1327 19696750 10.1038/nprot.2009.117

[33] Sauvinet V, Gabert L, Qin D, Louche-Pélissier C, Laville M, Désage M (2009) Validation of pentaacetylaldononitrile derivative for dual 2H gas chromatography/mass spectrometry and 13C gas chromatography/combustion/isotope ratio mass spectrometry analysis of glucose. Rapid Commun Mass Spectrom 23(23):3855–3867 19904737 10.1002/rcm.4294

[34] Reinauer H, Gries FA, Hübinger A, Knode O, Severing K, Susanto F (1990) Determination of glucose turnover and glucose oxidation rates in man with stable isotope tracers. J Clin Chem Clin Biochem 28(8):505–511 2258712 10.1515/cclm.1990.28.8.505

[35] Rowley NM, Madsen KK, Schousboe A, White HS (2012) Glutamate and GABA synthesis, release, transport and metabolism as targets for seizure control. Neurochem Int 61(4):546–558. 10.1016/j.neuint.2012.02.013 22365921 10.1016/j.neuint.2012.02.013

[36] Kanamori K (2017) Faster flux of neurotransmitter glutamate during seizure—evidence from ^13C-enrichment of extracellular glutamate in a rat model. PLoS ONE 12(4):e0174845. 10.1371/journal.pone.0174845 28403176 10.1371/journal.pone.0174845 PMC5389799

[37] During MJ, Spencer DD (1993) Extracellular hippocampal glutamate and spontaneous seizure in the conscious human brain. Lancet 26(8861):1607–1610. 10.1016/0140-6736(93)90754-5 10.1016/0140-6736(93)90754-5 8099987

