# Correction: Detection of regional metabolic alteration using 7T deuterium metabolic imaging in MRI-negative, ^18^FDG-PET-positive epilepsy patients

**DOI:** 10.1007/s10334-026-01360-9

**Published:** 2026-05-14

**Authors:** Narjes Ahmadian, Romy Buijs, Mark Gosselink, igrid Otto, Kiki Tesselaar, Jeanine J. Prompers, Pieter van Eijsden, Nicole van Klink, Dennis W. Klomp, Evita C. Wiegers

**Affiliations:** 1 Center for Image Sciences, University Medical Center Utrecht, Utrecht, Netherlands; 2 Department of Neurology and Neurosurgery, Brain Center, University Medical Center Utrecht, Utrecht, Netherlands; 3 CTI Lab Support, University Medical Center Utrecht, Utrecht, Netherlands; 4 Human Biology, NUTRIM School of Nutrition and Translational Research in Metabolism, Maastricht University Medical Center+, Maastricht, Netherlands

**Correction: Magnetic Resonance Materials in Physics, Biology and Medicine** 10.1007/s10334-026-01334-x

In the original version of this article, the superscripts in the abstract in 2H, all were written wrong.

The abstract should have read

**Abstract**

**Objective** Identifying presumed epileptogenic region (PER) in MRI-negative epilepsy patients is crucial for successful surgical outcomes. This study aims to determine whether dynamic deuterium metabolic imaging (DMI) at 7Tesla can detect region-specific metabolic alterations in MRI-negative, ^18^FDG-PET-positive temporal-lobe epilepsy.

**Methods** Five drug-resistant MRI-negative, ^18^FDG-PET-positive epilepsy patients underwent dynamic DMI. 3D ^2^H FID-MRSI scans (11:44 min each) were acquired at 7T over ~ 100 min following oral [6,6′-^2^H_2_]glucose intake. Venous plasma glucose and ^2^H glucose atom percent enrichment (APE) were measured in blood samples taken during scanning. Plasma glucose and ^2^H-glucose APE were analyzed using a General Linear Model with Repeated measures. From DMI, brain ^2^H-glucose and ^2^H glutamate/glutamine levels were analyzed using a two-level linear mixed model (factors: time and tissue type) comparing the PER and contralateral (contra-PER), hippocampus and temporal pole regions.

**Results** Plasma glucose and ^2^H-Glucose (Glc) atom-percent excess rose within 40 min post ingestion and then stabilized. Brain ^2^H-Glc and ^2^H-glutamate/glutamine (Glx) increased over time (p < 0.001). For ^2^H-Glc, regional differences were small, with only a modest elevation in hippocampus-PER relative to temporal-contra (p = 0.04). In contrast, ^2^H-Glx showed clear regional variation (p < 0.001), with the highest levels in hippocampus-PER, significantly exceeding PER, Temporal-PER, and Temporal-Contra (p ≤ 0.01). These findings remained consistent when averaging the final four time points.

**Conclusions** Ultra-high-field DMI revealed elevated hippocampal glutamatergic turnover in MRI negative epilepsy patients, with the highest levels in the epileptogenic hippocampus. These findings indicate the presence of subtle metabolic alterations in hippocampal tissue, supporting the potential of DMI to capture pathophysiological changes that remain invisible to conventional imaging.

The original article has been corrected.

