# Supplementary figures and images for: Detection of regional metabolic alteration using 7T deuterium metabolic imaging in MRI-negative, 18FDG-PET-positive epilepsy patients
Source: MAGMA. 2026 Feb 19;39(3):453–61. doi: 10.1007/s10334-026-01334-x (PMC13354614; doi:10.1007/s10334-026-01334-x)

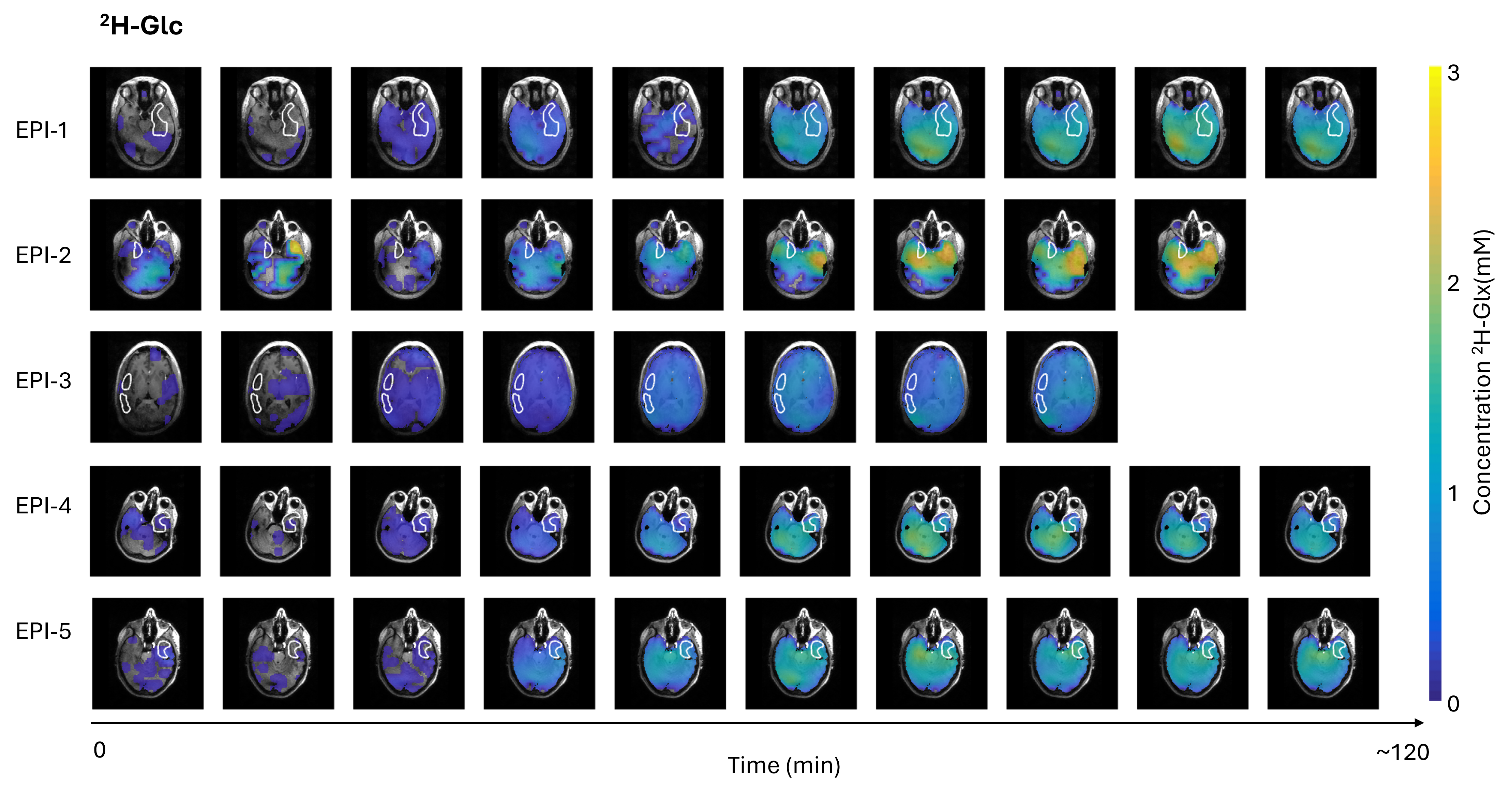

Supplement: Supplementary file 1 — Dynamic DMI maps of 2H-Glc after oral consumption of [6,6′-2H2]glucose, per subject (Epi-1 to Epi-5), including of all timepoints. The presumed epileptogenic region (PER) is indicated by the outlined areas. Color bars indicate metabolite concentrations (in mM). Supplementary file1 (PNG 4791 KB) [file 10334_2026_1334_MOESM1_ESM.png]

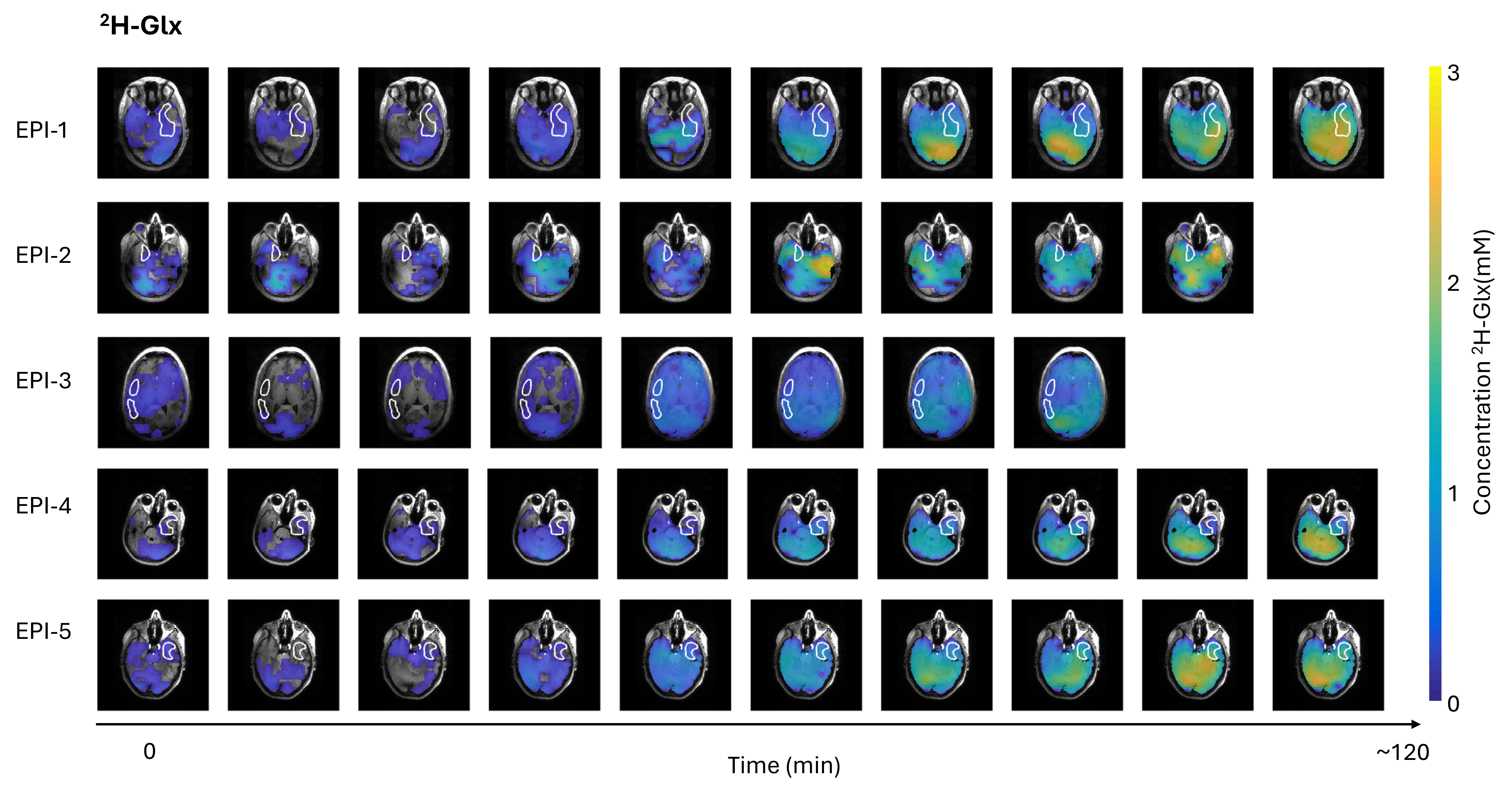

Supplement: Supplementary file 2 — Dynamic DMI maps of 2H-Glx after oral consumption of [6,6′-2H2]glucose, per subject (Epi-1 to Epi-5), including of all timepoints. The presumed epileptogenic region (PER) is indicated by the outlined areas. Color bars indicate metabolite concentrations (in mM). Supplementary file2 (PNG 4818 KB) [file 10334_2026_1334_MOESM2_ESM.png]

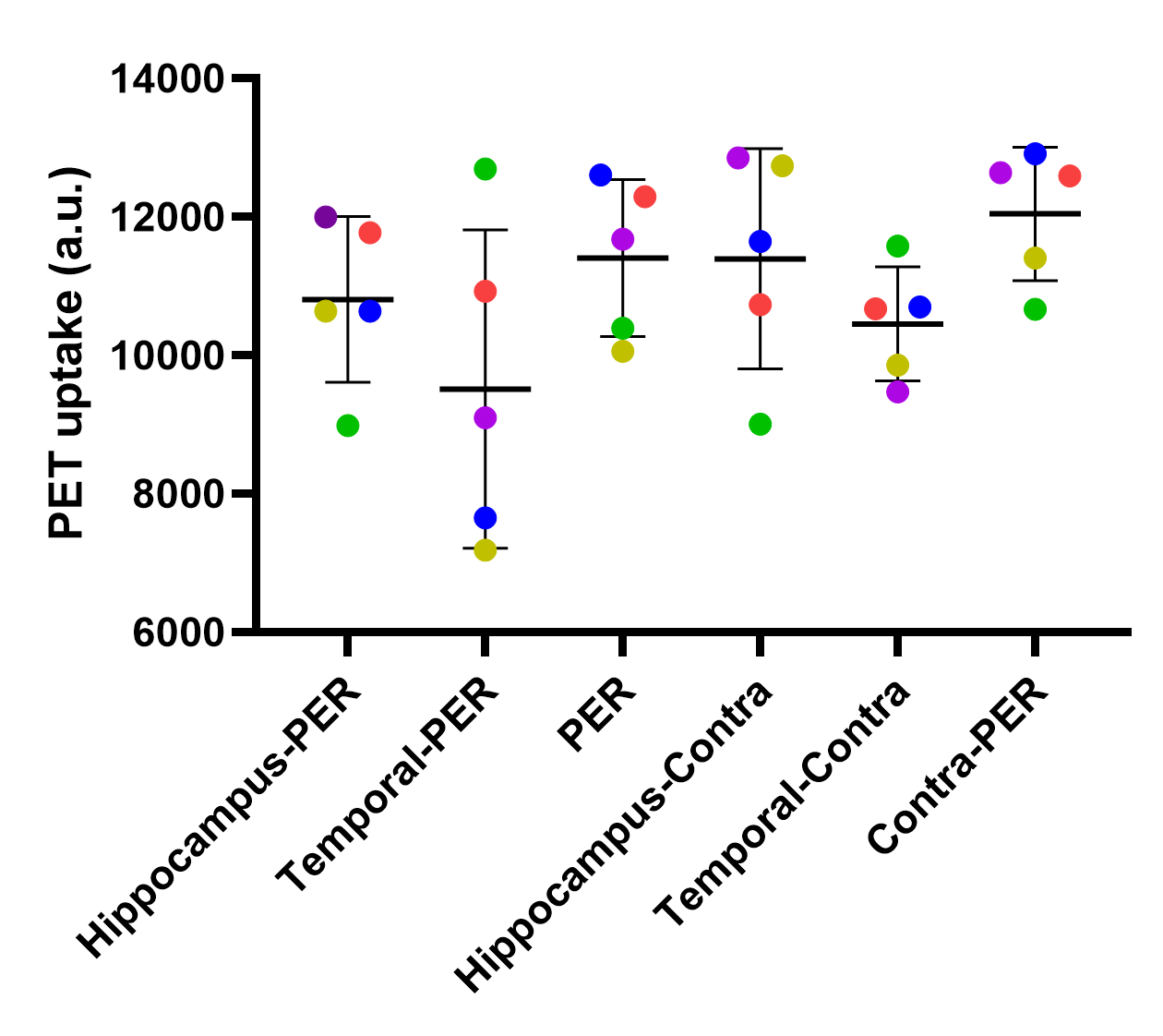

Supplement: Supplementary file 3 — Mean 18FDG-PET uptake values across patients for the epileptogenic region (PER), hippocampus, and temporal pole, along with their contralateral side. Each dot represents an individual patient, with horizontal bars indicating group means and error bars showing standard deviations. Supplementary file3 (PNG 64 KB) [file 10334_2026_1334_MOESM3_ESM.png]

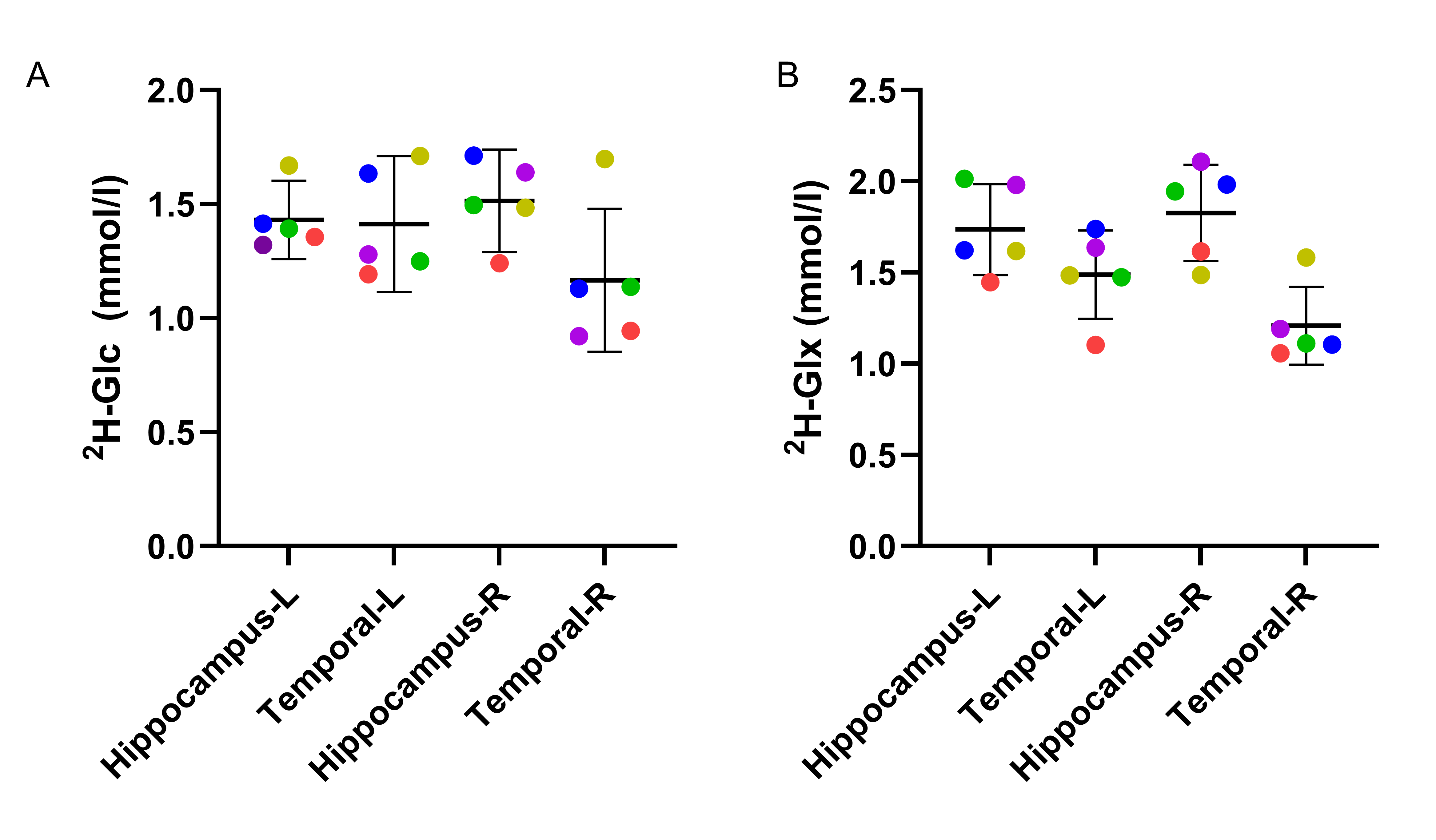

Supplement: Supplementary file 4 — Mean brain 2H-Glc (A) and 2H-Glx (B) levels over the final four time points in healthy volunteers. Metabolite concentrations are shown for the left and right hippocampus and temporal pole. Individual colors represent separate volunteers. Supplementary file4 (PNG 232 KB) [file 10334_2026_1334_MOESM4_ESM.png]
